# Association entre le niveau d'activité physique, l'indice de masse corporelle et la masse grasse chez des jeunes scolarisés dans la Wilaya de Marrakech (Maroc)

**DOI:** 10.11604/pamj.2020.35.78.13520

**Published:** 2020-03-19

**Authors:** Abdelmoujoud El Haboussi, Mohamed-Kamal Hilali, Mohamed Loukid

**Affiliations:** 1 Laboratoire d'Ecologie Humaine, Faculté des Sciences Semlalia, Université Cadi Ayyad, Marrakech, Maroc

**Keywords:** Activité physique, indice de masse corporelle, masse grasse, jeunes scolarisés, Marrakech, Al Haouz, Maroc, Physical activity, body mass index, body fat mass, young people of school age, Marrakech, Al Haouz, Morocco

## Abstract

**Introduction:**

L'activité physique est un bon indicateur de l'état de santé et du bien-être. Le présent travail se propose d'évaluer le niveau d'activité physique et d'étudier son association avec l'indice de masse (IMC) corporelle et la masse grasse chez des jeunes scolarisés dans la Wilaya de Marrakech.

**Méthodes:**

Un échantillon de 1173 jeunes scolarisés âgés de 15 à 20 ans a été constitué dans la ville de Marrakech (zone urbaine) et dans la province d'Al Haouz (zone rurale). Le niveau d'activité physique a été déterminé en mesurant la dépense énergétique par rapport à la dose d'activité physique recommandée. La régression logistique a été utilisée dans l'étude statistique des associations.

**Résultats:**

Quarante et un virgule un pourcent (41,1%) des enquêtés sont actifs pendant trois heures et plus par semaine, 31,1% pratiquant moins de trois heures par semaine, tandis que 27,3% (41,1% des filles contre 14,2% des garçons; p < 0,001) ne pratiquent aucune activité sportive de loisir en dehors de l'école. Les garçons sont plus actifs par rapport aux filles (OR = 3,16; p < 0,001). Les élèves ruraux sont plus actifs par rapport aux citadins (OR = 1,9; p < 0,001). Chez les filles, en passant du niveau «Sédentaire» au niveau «Très Actif», les moyennes de l'IMC et de la masse grasse ont diminué, respectivement, de 1,9 kg/m^2^ (F = 8,03; p < 0,001) et de 6,28% (F = 15,80; p < 0,001). Chez les garçons, la diminution était de 0,85 kg/m^2^ (F = 1,17; p = 0,17) pour l'IMC, et de 2,77% (F = 5,15; p = 0,006) pour la masse grasse.

**Conclusion:**

L'activité physique est significativement associée à la masse grasse et à l'IMC. Promouvoir l'activité physique des jeunes, dans leurs activités quotidiennes ainsi que pendant leurs loisirs, reste une première nécessité pour faire face aux effets néfastes d'un mode de vie sédentaire sur leur santé.

## Abstract (en)

**Introduction:**

Physical activity is a good indicator of health state and well-being. The purpose of this study is to assess physical activity level and to study its association with body mass index and body fat mass in young people of school age in the Wilaya of Marrakech.

**Methods:**

A sample of 1173 young people of school age (aged 15- 20 years) was recruited in the city of Marrakech (urban area) and in the Al Haouz Province (rural area). Physical activity level was determined by measuring the energy expenditure compared to the recommended levels of physical activity. Logistic regression was used in the statistical study of these associations.

**Results:**

Forty-one point one percent (41.1%) of respondents were physically active for three hours and more per week, 31.1% practiced less than three hours per week, while 27.3% (41.1% of girls against 14.2% of boys; p < 0.001) do not practice any sport activity in their free time outside the school. Boys were more active than girls (OR = 3.16; p < 0.001). Rural students were more active than urban dwellers (OR = 1.9; p < 0.001). Among girls, passing from the level of “sedentary” lifestyle to the level “very active” lifestyle, the means of body-mass index (BMI) and body fat mass decreased by 1.9 kg/m^2^ (F = 8.03; p < 0.001) and 6.28% (F = 15.80; p < 0.001) respectively. Among boys, the decrease in BMI was 0.85 kg/m^2^ (F = 1.17; p = 0.17) and 2.77% (F = 5.15; p = 0.006) in body fat.

**Conclusion:**

Physical activity is significantly associated with body fat mass and BMI. Physical activity promotion in young people into their daily activities as well as during their leisure time, is a basic need to cope with the downstream effects of sedentary lifestyle on their health.

## Introduction

Le mode de vie actuel, très mécanisé et très automatisé, associé à l'évolution de plus en plus croissante des technologies de l'information et de la communication, se caractérise par une réduction marquée du rôle de la musculature humaine. De nombreuses études ont mis en évidence les bénéfices physiologiques et psychologiques de la pratique d'une activité physique (AP) régulière et, a contrario, les conséquences néfastes de la sédentarité sur la santé [1-4]. A l'échelle internationale, les études sur ce sujet ont pris plus d'importance, en particulier après l'extension rapide et préoccupante des maladies non transmissibles liées à l'évolution de modes de vie et aux désordres métaboliques engendrés, à savoir l'obésité, la dyslipidémie, l'hypertension artérielle, le diabète de type II et le stress. Toutefois, à l'heure actuelle, les études consacrées à ce sujet au Maroc, et en particulier auprès des jeunes scolarisés, sont très restreintes voire inexistantes. La région de Marrakech-Safi est parmi celles dont nous avons noté le manque de ce genre de données. La présente étude vient pour combler ce manque dans les données nationales et pour enrichir celles disponibles à l'échelle du continent africain. Elle propose, dans un premier temps, de décrire l'AP et certaines caractéristiques anthropométriques, puis dans un deuxième temps, d'analyser l'association entre le niveau d'activité physique (NAP), l'indice de masse corporelle (IMC) et la masse grasse (MG) auprès des jeunes scolarisés issus de la ville de Marrakech (milieu urbain) et de la province d'Al-Haouz (milieu rural) au Maroc.

## Méthodes

### Zone et cadre de l'étude

Avec une superficie de 39167 km^2^, soit 5,5% du territoire national, la région de Marrakech-Safi se situe au centre du pays et englobe une partie du Haut-Atlas. Elle est composée de la préfecture de Marrakech et de 7 provinces (*Al-Haouz, Chichaoua, El-Kelâa des Sraghna, Essaouira, Rehamna, Safi et Youssoufia*). D'après le Haut-Commissariat au Plan (HCP), la population de la région représente 13,4% de la population nationale. Le taux d'urbanisation est de l'ordre de 42,8% [5]. La ville de Marrakech, chef-lieu de la région, représente en grande partie son milieu urbain avec un taux d'urbanisation de 73,6% [5]. La province d'Al-Haouz, conserve sa nature rurale dans sa grande partie avec un taux d'urbanisation qui ne dépasse pas 14,7% [5]. Dans le cadre d'un projet de recherche sur l'AP et l'état de santé des jeunes scolarisés, nous avons mené une enquête transversale en 2015, auprès des jeunes adolescents dans la ville de Marrakech (milieu urbain) et la province d'Al-Haouz (milieu rural). L'échantillon, formé de 1173 individus âgés de 15 à 20 ans, a été constitué dans certains établissements scolaires publics de la zone d'étude. L'enquête est basée sur un questionnaire permettant la collecte d'informations détaillées sur le mode de vie en général et sur l'AP habituelle en particulier. Nous avons relevé pour chaque adolescent inclus dans l'étude les mensurations anthropométriques de base (poids, taille, masse grasse).

### Activité physique et dépense énergétique

L'activité physique (AP) est définie comme la somme, durant un temps donné (une semaine ou une journée), des situations nécessitant la mise en jeu de la musculature squelettique avec augmentation de la dépense énergétique par rapport aux conditions de repos [6]. Nous avons considéré 4 grands domaines de la vie courante (domicile, transport, école, sport et loisirs) auxquels correspondent 4 catégories d'AP: 1) AP domestique: toute activité physique, autre que l'activité principale, exercée dans le cadre du foyer: tâches ménagères (vaisselle, repassage, nettoyage des surfaces, rangement); bricolage, jardinage, préparation de repas, ou autre tâche ou activité dans le cadre du foyer; 2) AP de transport: représente le mode principal utilisé pour fréquenter l'école, soit le mode actif (la marche à pied et/ou le vélo) ou le mode passif (moto, bus, taxi, voiture,"*Coutchi*", etc.); 3) AP scolaire: toute activité physique ou sportive pratiquée dans le cadre scolaire; nous distinguons entre les cours de l'éducation physique (EP) comme matière d'enseignement scolaire et les séances du Sport Scolaire (SS) dispensées au sein de l'Association Sportive Scolaire (ASS); 4) AP de sport et loisirs: comporte des activités physiques encadrées (dans des institutions sportives: un club ou une association sportive) et des activités physiques pratiquées librement sans encadrement.

Nous avons relevé, pour chaque domaine, les caractéristiques de l'AP (la durée, l'intensité et la fréquence hebdomadaire). L'intensité est exprimée en équivalent métabolique de la tache par rapport au repos MET (“Metabolic Equivalent of Task”), les valeurs considérées dans les calculs correspondent à celles du compendium des activités physiques [7]. La dépense énergétique liée à l'activité physique (DEAP) a été calculée selon la formule suivante:

DEAP = Σ (D x Fr x I)_n_

Où:

D: la durée (en heures) par séance, consacrée à l'AP (par domaine); Fr: la fréquence hebdomadaire (nombre de fois par semaine); I: l'intensité de l'AP d'après le compendium des activités physiques [7]; n: l'indice du domaine de l'AP (de 1 à 4).

Cette formule a été inspirée des études publiées [8, 9]. L'AP dont l'intensité est strictement inférieure à 1,6 MET est considérée comme activité sédentaire [10] et de ce fait a été exclue des calculs. Selon les recommandations internationales [11, 12], la dose journalière d'AP préconisée pour les jeunes (enfants et adolescents) est l'accumulation de 60 min (1 heure) d'AP modérée chaque jour, soit 7 heures par semaine. L'AP modérée est l'équivalent d'une dépense énergétique de 4 MET [13]. La valeur de DEAP 28 MET correspond à la dose recommandée par semaine. Dans la présente étude nous avons considéré comme «Sédentaire», le sujet qui ne cumule pas 14 MET (soit l'équivalent de la moitié de la dose recommandée), il est considéré «Actif», tout sujet cumulant au moins 14 MET sans atteindre 28 MET alors que le sujet qui atteint ou dépasse 28 MET est considéré comme étant «Très Actif».

### Paramètres socioéconomiques

Dans cette étude, nous avons considéré les paramètres suivants: la classe socioprofessionnelle (CSP) du chef de ménage selon la proposition de Orban-Seghebarth et collaborateurs [14], nous distinguons la CSP1 (grands commerçants et professions libérales); la CSP2 (fonctionnaires et cadres); la CSP3 (artisans, salariés, ouvriers, agriculteurs, chauffeurs, aides commerçant); et la CSP4 (personnes sans profession); le niveau de formation ou le dernier niveau d'instruction atteint par le chef de ménage; nous distinguons ainsi, le niveau 1 (Néant ou Fondamental), le niveau 2 (Secondaire ou Technique) et le niveau 3 (Supérieur); la structure familiale; nous distinguons la famille nucléaire (un seul couple dans la famille) et la structure multiple (plus d'un couple); la fratrie: nombre de frères et sœurs.

### Mensurations et indicateurs anthropométriques

Les mensurations anthropométriques ont été prises à l'aide du matériel et de procédures valides. La taille et le poids de chaque sujet ont été mesurés selon la procédure recommandée en vêtements d'intérieur, sans les chaussures. Le poids a été mesuré à l'aide d'une balance pèse-personne marque seca, avec une précision de 0,1 kg; la taille a été mesurée à l'aide d'une toise anthropométrique démontable avec une précision de 0,1 cm, et la MG a été déterminée par l'impédance-mètre bioélectrique à 4 pôles (Tanita BC 545N).

### Méthode d'échantillonnage

Aucun caractère d'exclusion n'a été posé, tous les enfants et adolescents inscrits dans les établissements scolaires appartenant à la zone d'étude ont eu la même chance d'être inclus dans l'échantillon. Nous avons choisi au hasard 8 établissements parmi les 32 lycées situés au centre de la ville (milieu urbain). Pour le milieu rural (la province d'Al-Haouz), le choix de 5 établissements a été fait au hasard parmi les 10 lycées situés dans les cinq cercles administratifs de la province (Ait ourir, Amizmiz, Asni, Tahannaout et Touama). Dans chaque établissement, les individus ont eu la même chance d'être choisis.

**Analyse et traitement statistique:** l'analyse statistique des données a été réalisée à l'aide du logiciel SPSS version 20 (IBM^®^ SPSS Statistics^®^). Pour les données qualitatives, les comparaisons des fréquences et des pourcentages ont été effectuées par le test statistique de Khi-2. Les moyennes des paramètres quantitatifs ont été comparées à l'aide de test paramétrique de comparaison de moyennes (test t de Student) ou à l'aide de test ANOVA s'il y a plus de deux moyennes à comparer. Sauf précision, les données sont exprimées en moyenne ± écart-type ou pourcentage, selon le type de données. Le seuil de signification statistique pour tous les tests a été fixé à p=0,05. Des modèles de régression logistique ont été réalisés pour examiner l'association entre le niveau d'activité physique et certains facteurs socioéconomiques des familles.

### Considérations éthiques

Pour respecter les considérations éthiques, nous assurons la confidentialité et le respect de tous lors de cette recherche après avoir expliqué aux participants les objectifs de l'étude et leurs implications. Ainsi l'enquête s'est déroulée sous forme d'une interview avec chaque élève individuellement, le principe de volontariat pour la participation ainsi que l'anonymat du questionnaire ont été respectés. Une autorisation écrite a été fournie par la Direction de l'Académie Régionale de l'Education et la Formation de Marrakech-Safi avant d'entamer l'enquête au sein des établissements scolaires.

## Résultats

### L'échantillon

L'échantillon est constitué de 1173 enfants et adolescents scolarisés dont 48,3% filles. Il est réparti sur les deux milieux, rural et urbain, 56,9% sont issus du milieu rural contre 42,3% qui sont issus du milieu urbain. L'âge des participants varie de 15 à 20 ans avec une moyenne de 17,54±1,59 ans (Tableau 1).

**Tableau 1:** La moyenne d’âge et la répartition de l’échantillon par milieu de résidence et par sexe

		Sexe	Moyenne	Ecart-type	N	% du total
**Milieu**	**Rural**	Masculin	17,85	1,58	355	56,9%
		Féminin	17,37	1,30	312	
		Total	17,62	1,47	667	
	**Urbain**	Masculin	17,44	1,81	252	43,1%
		Féminin	17,44	1,62	254	
		Total	17,44	1,72	506	
**Total**		Masculin	17,68	1,69	607	51,7%
		Féminin	17,40	1,45	566	48,3%
		Total	17,54	1,59	1173	100%

La différence de moyenne d’âge est non significative par rapport au milieu de résidence

### Description de l'activité sportive auprès des jeunes scolarisés

**Figure 1:**
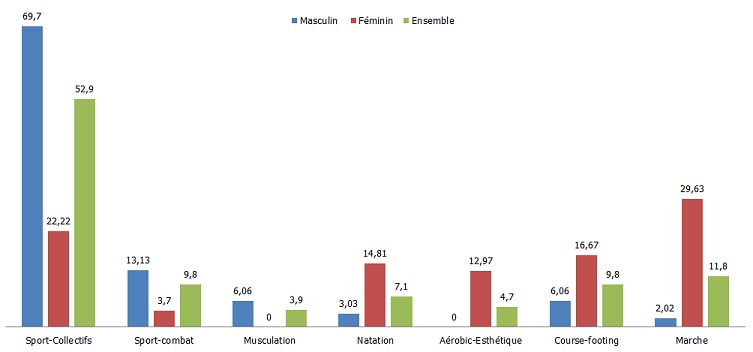
Le type d'activité sportive pratiquée principalement comme loisir

Près de 73% des lycéens enquêtés ont déclaré avoir une activité sportive de loisir (ASL) pratiquée habituellement en dehors de l'établissement scolaire (Tableau 2). Près de 41% d'entre eux sont actifs au moins pendant trois heures par semaine (soit l'équivalent de 60 min/jour avec une fréquence de 3 fois par semaine), 31,1% semble avoir une ASL mais avec une dose relativement moins importante (moins de trois heures par semaine). Vingt-sept virgule trois pourcent (27,3%) des lycéens ne pratiquent aucune ASL en dehors de l'établissement scolaire. La proportion des filles qui ne font pas d'ASL est presque trois fois plus que celle des garçons (41,1% des filles contre 14,2% des garçons). Les jeunes scolarisés dans le milieu rural semblent être plus actifs que leurs homologues citadins. En effet, près de 45% d'entre eux ont déclaré être pratiquants d'une ASL d'au moins trois heures par semaine contre 37% en milieu urbain (Tableau 2). Quant au type d'ASL pratiquée (Figure 1), au total, les sports collectifs (football, basketball, volleyball et handball) viennent en premier avec un pourcentage de 52,9%, suivis de la marche (11,8%), puis des sports de combat (karaté, taekwondo) et des courses qui viennent ensemble en 3^e^ place (9,8% chacun). Les résultats montrent également que le type d'ASL dépend du sexe. On retrouve une proportion plus élevée de garçons par rapport aux filles pour les sports collectifs, les sports de combat et la musculation. Et l'inverse pour les autres activités, à savoir, la marche, la course-footing, la natation, la danse et l'aérobic qui sont préférées par les filles.

**Tableau 2:** La répartition des élèves en effectifs et en pourcentage selon la durée hebdomadaire consacrée à la pratique des activités physiques de loisirs selon le sexe et le milieu de résidence

	Sexe		Milieu		Total	
	Masculin	Féminin	Rural	Urbain		
	N (%)	N (%)	N (%)	N (%)	N (%)	% cumulé
**3h et plus**	339 (58,0)	134 (24,3)	294 (45,2)	179 (36,9)	473 (41,6)	41,1
**Moins de 3h**	162 (27,7)	191 (34,6)	169 (26,0)	184 (37,9)	353 (31,1)	72,7
**Aucune ASL**	83 (14,2)	227 (41,1)	188 (28,9)	122 (25,2)	310 (27,3)	100
**Test Khi-2**	157,34; p < 0,001		18,79; p<0,05			

Les résultats sur le Tableau 3 montrent qu'environ 55% des enfants et adolescents scolarisés sont « Actifs » et seulement environ 10% qui sont classés « Très Actifs », alors que 34,4% sont « Sédentaires ». Le NAP, cependant, varie selon le sexe: les filles sont moins actives par rapport aux garçons: 44% des filles sont classées aux NAP « Sédentaire » contre 23,5% pour les garçons; alors que les garçons sont classés aux NAP « Très Actif » plus que les filles (soit près de 18% contre 3,3%). D'autre part le NAP varie selon le milieu de résidence. Dans l'ensemble, la proportion des élèves résidant en milieu rural et classés au niveau « Très Actif », est presque deux fois plus élevée que celle des élèves résidant en milieu urbain (12% contre 7,5%). A l'aide de modèles de régression logistique tenant compte de différents facteurs de confusion (Tableau 4), nous avons étudié le NAP des jeunes scolarisés en fonction du sexe, du milieu de résidence et certains facteurs socioéconomiques de leurs familles, à savoir la CSP et le niveau de formation du chef de ménage, la structure familiale et le nombre de frères et sœurs. Il en ressort que le sexe masculin est actif par rapport au féminin (OR: 3,16). Egalement, lorsque le jeune est issu du milieu rural, il est susceptible d'être plus actif par rapport au jeune issu du milieu urbain (OR: 1,9). Le modèle de régression logistique n'a pas montré d'association statistiquement significative entre le niveau d'activité physique et les autres facteurs socioéconomiques étudiés.

**Tableau 3:** La répartition des élèves en effectifs et en pourcentage selon le niveau d'activité physique, le sexe et le milieu de residence

		Milieu		Total N (%)
	Niveau d’AP	Rural N (%)	Urbain N (%)	
**Garçons**	**Sédentaire**	63 (19,5)	53 (31,2)	116 (23,5)
	**Actif**	199 (61,6)	90 (52,9)	289 (58,6)
	**Très actif**	61 (18,9	27 (15,9)	88 (17,8)
	**Test Khi-2**	8,43; p=0,015		
**Filles**	**Sédentaire**	101 (33,2)	141 (57,6)	242 (44,1)
	**Actif**	189 (62,2)	100 (40,8)	289 (52,6)
	**Très actif**	14 (4,6)	4 (1,6)	18 (3,3)
	**Test Khi-2**	33,62; p<0,001		
**Ensemble**	**Sédentaire**	164 (26,2)	194 (46,7)	358 (34,4)
	**Actif**	388 (61,9	190 (45,8)	578 (55,5)
	**Très actif**	75 (12,0)	31 (7,5)	106 (10,2)
	**Test Khi-2**	47,43; p<0,001		

**Tableau 4:** L'association entre l'activité physique (non actif, actif), le milieu de résidence et certains facteurs socioéconomiques des ménages (régression logistique)

		Activité physique (Non Actif/Actif)		
		n=1173		
**Variables prédictives**		**OR**	**IC 95%**	**p**
**Sexe**	Masculin	3,16	2,36-4,21	0,00
	Féminin	.	.	.
**Milieu**	Rural	1,90	1,43-2,53	0,00
	Urbain	.	.	.
**Niveau de formation^(1)^**	Néant/fondamental	1,12	0,73-1,73	0,60
	Secondaire/technique	1,22	0,77-1,94	0,40
	Supérieur	.	.	.
**Classe Socioprofessionnelle^(2)^**	CSP1	1,15	0,46-2,89	0,76
	CSP2	0,89	0,39-2,01	0,78
	CSP3	1,01	0,48-2,12	0,99
	CSP4	.	.	.
**Structure familiale**	Nucléaire	0,77	0,52-1,14	0,19
	Multiple	.	.	.
**Nombre de frère et sœurs**		0,97	0,89-1,06	0,46

(1) niveau de formation ou dernier niveau d’instruction du chef de ménage

(2) Classe socioprofessionnelle du chef de ménage

### Caractéristiques anthropométriques par sexe et par milieu de résidence

D'après les résultats du Tableau 5, nous remarquons que, chez les garçons, l'IMC et la MG sont significativement plus importants en milieu urbain par rapport au milieu rural soit respectivement 20,79 kg/m^2^ contre 19,98 kg/m^2^ et 14,78% contre 13,59%, tandis que nous n'avons noté aucune différence significative ni en taille, ni en poids, entre le milieu urbain et le milieu rural. Chez les filles, tous les indicateurs biométriques étudiés sont plus importants en milieu urbain par rapport au milieu rural, soit, la taille (161,14m contre 159,67m), le poids (55,25kg contre 52,68kg), l'IMC (21,36 kg/m^2^ contre 20,73 kg/m^2^) et la MG (23,68% contre 20,77%), ces différences observées sont statistiquement significatives (Test t de Student porté sur le Tableau 5).

**Tableau 5:** Les caractéristiques anthropométriques par sexe et par milieu de résidence

Sexe	Caractéristiques	Milieu	N	Moyenne	Ecart-type	Test t
**Garçons**	**Taille (cm)**	Rural	341	171,29	7,50	1,48; ddl=274 p = 0,139: N.S
		Urbain	183	169,95	11,01	
	**Poids (kg)**	Rural	339	58,71	9,60	-1,63; ddl=312 p = 0,104: N.S
		Urbain	181	60,35	11,65	
	**IMC**	Rural	338	19,98	2,72	-2,63; ddl=284 p = 0,009: **
		Urbain	178	20,79	3,62	
	**Masse Grasse (%)**	Rural	338	13,59	5,81	-2,19; ddl=513 p = 0,028: *
		Urbain	177	14,78	5,94	
**Filles**	**Taille (cm)**	Rural	292	159,67	6,36	-2,34; ddl=476 p = 0,02: *
		Urbain	186	161,14	7,25	
	**Poids (kg)**	Rural	298	52,68	7,73	-3,44; ddl=469 p = 0,001: **
		Urbain	173	55,25	7,98	
	**IMC**	Rural	291	20,73	2,68	-2,41; ddl=459 p = 0,016: *
		Urbain	170	21,36	2,76	
	**Masse Grasse (%)**	Rural	285	20,77	6,77	-5,36; ddl=437 p < 0,001: **
		Urbain	168	23,68	4,75	

* p<0,05: significatif

** p<0,001: très significatif; N.S: non significatif

### Association entre le NAP, l'IMC et la masse grasse

**Figure 2:**
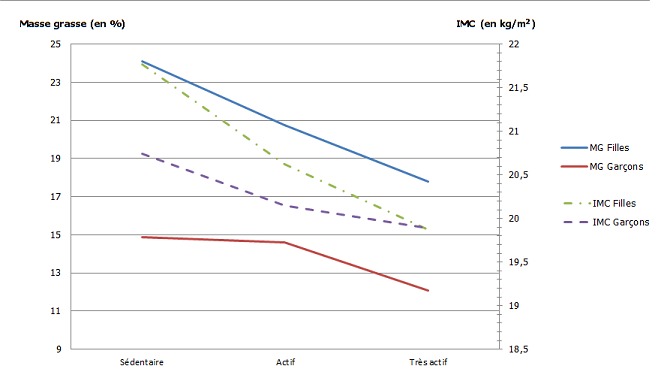
Évolution de la moyenne de la masse grasse et de l'IMC en fonction du niveau d'activité physique

Les résultats illustrés par la Figure 2 montrent qu'indépendamment du sexe, l'IMC est associé avec le NAP. Plus le NAP est élevé plus la moyenne de l'IMC est réduite. Chez les filles, entre le groupe « Sédentaire » et celui qui est « Très Actif », la différence de moyenne de l'IMC était de 1,9 kg/m^2^ (Test ANOVA: F = 8,03; p < 0,001; très significatif). Chez les garçons, la différence était de 0,85 kg/m^2^ (Test ANOVA: F = 1,17; p = 0,17; non significatif). Le pourcentage de la graisse corporelle (% MG) est significativement réduit lorsque le NAP est élevé (Figure 2). La réduction de la MG en fonction du NAP est plus marquée chez les filles par rapport aux garçons. En effet, la différence de moyenne entre le groupe « Sédentaire » et celui qui est « Très Actif », était 6,28% chez les filles (Test ANOVA: F = 15,80; p < 0,001) et 2,77% chez les garçons (Test ANOVA: F = 5,15; p = 0,006).

## Discussion

Les résultats de la présente étude ont montré qu'environ 27% des enfants scolarisés enquêtés ont déclaré n'avoir aucune ASL. Ce pourcentage est deux fois plus élevé que celui d'un groupe d'enfants/adolescents scolarisés français, dont seulement 14% ayant déclaré n'avoir jamais pratiqué de sport en dehors de l'établissement scolaire [15]. Près de 41% des scolarisés enquêtés sont actifs au moins pendant trois heures par semaine. Seulement 10,2% d'entre eux qui sont classés au NAP « Très Actif », c'est la proportion des jeunes scolarisés qui sont suffisamment actifs par rapport aux recommandations internationales [11] cette proportion permet de situer ces jeunes scolarisés en dessous des limites des recommandations internationales.

Les résultats ont montré également que le NAP est significativement associé au milieu de résidence. Malgré que les élèves citadins aient plus de chance de pratiquer des ASL variées et structurées dans les clubs et/ou les associations sportives, les élèves en milieu rural sont plus actifs dans leur vie courante. Ils pratiquent davantage de loisirs dans un cadre non structuré, ils exercent plus d'activités dans le cadre domestique (travail aux champs, aide familial, …etc.) et ils adoptent le plus souvent un transport actif (le vélo et la marche) comme mode principal pour rejoindre l'établissement scolaire. En plus, les établissements scolaires en milieu rural sont le plus souvent situés loin de l'habitat des élèves ceci augmentera absolument leur DEAP et par conséquent leur NAP. La pratique d'AP est également significativement liée au sexe, nous avons noté que les garçons en général sont plus actifs que les filles. Ce résultat est en accord avec les données de l'enquête HBSC (Health Behaviour in School-aged Children) de l'OMS, où les filles, sont systématiquement moins actives que les garçons. A 11 ans, 19% des filles sont suffisamment actives, contre 28% des garçons.

En outre, la prévalence de l'AP diminue avec l'augmentation de l'âge à l'adolescence [16-19]. Nous n'avons révélé aucune association statistiquement significative entre le niveau d'activité physique et l'environnement socioéconomique, alors que selon certaines études, la position socioéconomique est significativement associée à la pratique de l'AP et aux comportements sédentaires, notamment dans l'enquête HBSC [20]. A l'échelle internationale, cette relation n'est pas toujours significative [21, 22]. Les résultats contradictoires proviennent probablement des différences culturelles entre les pays mais également des méthodes utilisées pour mesurer l'AP.

Les indicateurs IMC et MG sont associés à la fois, avec le sexe et le milieu de résidence. Ils sont plus élevés chez les filles par rapport aux garçons et plus élevés en milieu urbain qu'en rural. Ces résultats concordent avec celles d'une étude similaire de Koutar *et al.* réalisée auprès des jeunes scolarisés dans la même zone de la Wilaya de Marrakech, où l'IMC est supérieur chez les élèves du milieu urbain à tout âge [23]. L'IMC et la MG sont significativement associé avec le NAP quel que soit l'environnement socioéconomique. Ces deux paramètres de corpulence fournissent, ensemble, une indication sur la quantité de tissu adipeux corporel qui est étroitement corrélée avec différentes maladies et problèmes de santé. En effet, plus la graisse est importante, plus le risque de développer un diabète de type II, une hypertension artérielle ou une maladie cardiaque coronarienne est élevé.

Nos résultats ont montré que plus le NAP augmente plus la MG diminue, ce qui est en accord avec les recherches internationales menées sur des enfants et des adolescents, qui ont montré aussi que les individus qui se livrent à une AP relativement importante ont moins de tissu adipeux que ceux qui ne font pas d'exercice [24, 25] et d'autres recherches qui ont montré qu'il existe un rapport évident entre l'AP intensive et la stabilité pondérale [26]. Les effets de l'AP sur le contrôle du poids passent à la fois par la dépense d'énergie au-dessus de la valeur de repos (consommation des réserves musculaires en glycogène pendant l'effort, et reconstitution de ces réserves après l'arrêt de l'effort) et par un meilleur contrôle des apports alimentaires entourant la pratique de l'AP. De façon générale, l'AP a des effets positifs sur le plan psychologique en améliorant l'humeur, la sensation de bien-être et l'estime de soi [27]. Cet effet participe à la limitation de la prise de poids au cours des années [28]. Il est préférable de prévenir le gain de poids, d'autant que le traitement de l'obésité est très difficile et que le maintien de poids après amaigrissement est souvent impossible. En effet, 40% des enfants et 70% des adolescents obèses le demeurent à l'âge adulte [29], de ce fait l'AP demeure une habitude à ancrer dans le mode de vie des jeunes.

En agissant à la fois sur le plan physiologique (la dépense énergétique de l'organisme), et sur le plan psychologique, l'AP impacte significativement les indicateurs anthropométriques à savoir le poids, l'IMC et la MG. En effet, l'excès de la masse corporelle est nocif pour l'organisme car il exerce une surcharge sur les articulations ainsi que sur les tissus et augmente le risque de maladies chroniques (maladies coronariennes, diabète de type II, l'hypertension artérielle). Dans notre étude, les jeunes scolarisés dans le milieu urbain sont plus lourds en IMC et MG que leurs homologues en milieu rural et de ce fait ils sont fort probablement plus exposés aux problèmes de santé au cours de leur vie. Le surpoids acquis au cours de l'enfance ou de l'adolescence peut persister à l'âge adulte et augmenter le risque d'avoir, plus tard au cours de la vie, des maladies liées à l'obésité et qui compromettraient le pronostic fonctionnel et vital des sujets affectés [30, 31]. A l'inverse, les personnes qui pratiquent régulièrement une AP avec une dose assez suffisante peuvent maintenir un poids normal et, en parallèle, atténuer le risque de développer des maladies chroniques, tout en préservant leurs indicateurs biométriques dans l'intervalle de valeurs saines.

Dans cette zone d'étude, les individus qui font moins d'AP, sont moins lourds et ont moins de graisse corporelle; néanmoins cette association ne peut être vue seulement dans un sens unidirectionnel qui stipule que l'AP fait diminuer l'IMC et la MG, mais aussi il se peut que les sujets en surpoids font souvent moins d'exercice physique. De ce fait, d'autres études doivent être menées pour confirmer le sens de ce lien et le degré de la relation de causalité. Notamment, un suivi de la relation cause à effet selon un programme d'exercice physique et la mesure de l'envie et de l'acceptabilité des sujets en surpoids à s'adonner aux exercices physiques.

## Conclusion

Dans l'ensemble, les garçons sont plus actifs que les filles, de même que le sont les adolescents scolarisés en milieu rural par rapport à leurs homologues en milieu urbain. Indépendamment du milieu de résidence et des conditions socioéconomiques, le NAP est significativement associé aux indicateurs de corpulences (IMC et MG), dans le sens qu'un NAP élevé aide à maintenir un statut pondéral sain et à réduire le tissu adipeux, et de ce fait, l'AP impacte positivement l'état de santé. Pour augmenter le NAP, l'AP devrait être d'une part, une routine quotidienne de chacun, à domicile, à l'école et dans ses déplacements, et d'autre part la base des loisirs régulièrement pratiqués. Une attention particulière doit être orientée vers les institutions sportives (les clubs, les associations et les maisons de la jeunesse) pour encourager l'encadrement des jeunes. Il semble aussi important de revoir les volumes horaires spécifiques aux cours de l'éducation physique et au sport scolaire. Cependant, vu les différences observées, entre les filles et les garçons d'une part et entre le milieu rural et urbain d'autre part, la mise en place des stratégies particulières selon le sexe et le milieu de résidence s'imposent comme une priorité des politiques de santé scolaire en particulier et de la santé publique en général.

### Etat des connaissances actuelles sur le sujet

- L’AP apporte un bénéfice certain pour la santé, c’est le constat général sur ce sujet qui est largement étudié à l'échelle internationale;
- Mais plusieurs études se limitent, dans leurs objectifs, aux activités sportives ou de loisir et certaines d’autre en ajoutent l’activité physique de déplacement.

### Contribution de notre étude à la connaissance

- L’étude apporte des données nouvelles sur un sujet encore moins étudié au Maroc et particulièrement dans la région de Marrakech;
- L’étude met en relief les disparités d’une part entre les deux sexes et d’autre part entre le milieu urbain et rural vis-à-vis de la pratique sportive;
- Pour pallier à la vulnérabilité des filles et du milieu urbain, une politique égalitaire est à mettre en place.

## Conflits d'intérêts

Les auteurs ne déclarent aucun conflit d'intérêts.
